# Short report: Introduction of chikungunya virus ECSA genotype into the Brazilian Midwest and its dispersion through the Americas

**DOI:** 10.1371/journal.pntd.0009290

**Published:** 2021-04-16

**Authors:** Elaine Cristina de Oliveira, Vagner Fonseca, Joilson Xavier, Talita Adelino, Ingra Morales Claro, Allison Fabri, Eduardo Marques Macario, Ana Elisa Viniski, Claudio Luis Campos Souza, Evanil Sebastiana Gomes da Costa, Claudia Soares de Sousa, Flávia Guimarães Dias Duarte, Arnaldo Correia de Medeiros, Carlos F. Campelo de Albuquerque, Rivaldo Venancio Cunha, Noely Fabiana Oliveira De Moura, Ana Maria Bispo de Filippis, Tulio de Oliveira, José Lourenço, André Luiz de Abreu, Luiz Carlos Junior Alcantara, Marta Giovanetti

**Affiliations:** 1 Laboratório Central de Saúde Pública do Estado de Mato Grosso, Cuiabá, Brazil; 2 Laboratório de Genética Celular e Molecular, Universidade Federal de Minas Gerais, Belo Horizonte, Minas Gerais, Brazil; 3 KwaZulu-Natal Research Innovation and Sequencing Platform (KRISP), College of Health Sciences, University of KwaZuluNatal, Durban 4001, South Africa; 4 Coordenação Geral dos Laboratórios de Saúde Pública/Secretaria de Vigilância em Saúde, Ministério da Saúde, Brasília, Distrito Federal, Brazil; 5 Laboratório Central de Saúde Pública, Fundação Ezequiel Dias, Belo Horizonte, Brazil; 6 Instituto de Medicina Tropical e Faculdade de Medicina da Universidade de São Paulo, São Paulo, Brazil; 7 Laboratório de Flavivírus, Instituto Oswaldo Cruz, Fundação Oswaldo Cruz, Rio de Janeiro, Brazil; 8 Secretaria de Vigilância em Saúde/Ministério da Saúde, Brasília, Distrito Federal, Brazil; 9 Secretaria de Saúde de Cuiaba, Mato Grosso, Brazil; 10 Organização Pan-Americana da Saúde/Organização Mundial da Saúde, Brasília-DF, Brazil; 11 Fundação Oswaldo Cruz, Bio-Manguinhos, Rio de Janeiro, Brasil; 12 Coordenacao Geral das Arboviroses, Secretaria de Vigilância em Saúde/Ministério da Saúde, Brasília, Distrito Federal, Brazil; 13 Department of Zoology, University of Oxford, Oxford, OX1 3PS, United Kingdom; DoD - AFHSB, UNITED STATES

## Abstract

Since introduction into Brazil in 2014, chikungunya virus (CHIKV) has presented sustained transmission, although much is unknown about its circulation in the midwestern states. Here, we analyze 24 novel partial and near complete CHIKV genomes from Cuiaba, an urban metropolis located in the Brazilian midwestern state of Mato Grosso (MT).

Nanopore technology was used for sequencing CHIKV complete genomes. Phylogenetic and epidemiological approaches were used to explore the recent spatio-temporal evolution and spread of the CHIKV-ECSA genotype in Midwest Brazil as well as in the Americas.

Epidemiological data revealed a reduction in the number of reported cases over 2018–2020, likely as a consequence of a gradual accumulation of herd-immunity. Phylogeographic reconstructions revealed that at least two independent introductions of the ECSA lineage occurred in MT from a dispersion event originating in the northeastern region and suggest that the midwestern Brazilian region appears to have acted as a source of virus transmission towards Paraguay, a bordering South American country.

Our results show a complex dynamic of transmission between epidemic seasons and suggest a possible role of Brazil as a source for international dispersion of the CHIKV-ECSA genotype to other countries in the Americas.

## Author summary

Since its introduction into Brazil in 2014, chikungunya virus (CHIKV) has presented sustained transmission, although much is unknown about its circulation in the midwestern states. Here, using a combined strategy of a mobile sequencing mission through this region, with genomic and epidemiological analysis we generated 24 novel partial and near complete CHIKV genome sequences by means of portable nanopore sequencing of chikungunya virus isolates obtained directly from clinical samples. Our findings reinforce that continued genomic surveillance strategies are needed to assist in the monitoring and understanding of arbovirus epidemics, which might help to attenuate public health impact of infectious diseases.

## Introduction

After the introduction of the Asian lineage of chikungunya virus (CHIKV) into the Americas in 2013, and subsequent detection of the East/Central/South African (ECSA) lineage in 2014 in Bahia state Northeast Brazil, more than 2.9 million infections have been reported in Brazil up to 2019 [1]. Common clinical manifestations of the disease include fever, muscle pain, rash and severe joint pain, which may last for months to years [2]. It is argued that 82% of infections caused by the ECSA lineage are symptomatic, and there are suggestions of lineage-specific infection outcomes [3]. The ECSA lineage seems to be the main genotype currently circulating in Brazil since its introduction in the northeastern region in 2014, despite detection of the Asian lineage in that same year in northern Brazil [4]. From previous studies addressing interregional dispersion of the virus [4], CHIKV outbreaks have been registered in northern, northeastern, southeastern and midwestern states of Brazil between 2016 and 2019 [5–8]. The number of probable cases reached 87,687 in 2018 and 132,205 in 2019, with the midwestern region accounting for the second largest number of cases (15.8%) registered in 2018 in Brazil [9]. Despite the large number of cases, much is unknown about the genomic diversity and evolution of CHIKV lineages currently circulating in the midwestern region of Brazil, as well as their dispersion dynamics in South American countries that border this Brazilian region. Thus, to investigate the genomic diversity and evolution of CHIKV, we analyzed 24 new CHIKV genomes generated by next generation sequencing, providing additional information on the introduction and spread of the ECSA lineage in Midwest Brazil as well as in the Americas.

## Materials and methods

### Ethics statement

This research was reviewed and approved by the Ethical Committee of the Pan American World Health Organization (No. PAHO-2016-08-0029) and the Brazilian Ministry of Health (MoH) as part of the arbovirus genomic surveillance efforts within the terms of Resolution 510/2016 of CONEP (Comissão Nacional de Ética em Pesquisa, Ministério da Saúde; National Ethical Committee for Research, Ministry of Health). Residual anonymized clinical diagnostic samples, with no or minimal risk to patients, were provided for research and surveillance purposes within the terms of Resolution 510/2016 of CONEP. Processing of human samples was approved and the need for participants consent was waived by the Institutional Review Board from the Fundação Oswaldo Cruz/Instituto Oswaldo Cruz (CEP/CAAE: 90249218.6.1001.5248; approval number 2.998.362).

### Sample collection

A total number of 24 serum samples from individuals presenting with symptoms compatible with arbovirus infection, with availability of epidemiological metadata, such as date of symptom onset, date of sample collection, sex, age and municipality of residence, were collected for molecular diagnostics by the Central Public Health Laboratory of Mato Grosso state, Midwest Brazil, during genomic surveillance activities of the project ZIBRA2 (https://www.zibra2project.org/).

### Viral RNA isolation and quantitative real-time RT-PCR

Serum samples were submitted to nucleic acid purification using the QIAmp Viral RNA Mini kit (Qiagen), following manufacturer’s recommendations. The CHIKV RNA detection by RT-qPCR was performed using a Real Time RT-qPCR protocol adapted from [10], using Promega GoTaq Probe 1-Step RT-PCR System Kit in Bioer LineGene 9660 equipment. Samples were selected for sequencing based on Ct-value <32 to maximize genome coverage of clinical samples by nanopore sequencing [11] (**Table 1**).

**Table 1:** Epidemiological data for the sequenced samples.

ID	Sample type	Date onset symptoms	Collection date	Age	Sex	State	Municipality	Ct value	Reads	Coverage (%)	Acession Number
CB08	Serum	2018-02-23	2018-02-23	33	F	MT	Cuiaba	18,59	900	43,70	MN428504
CB04	Serum	2018-05-18	2018-05-21	22	F	MT	Cuiaba	30,98	4318	52,60	MN428505
CB09	Serum	2018-01-29	2018-02-01	23	F	MT	Cuiaba	19,66	291904	93,60	MN428506
CB10	Serum	2018-07-03	2018-07-05	29	F	MT	Cuiaba	26,88	620828	82,70	MN428507
CB12	Serum	2018-04-25	2018-04-27	22	F	MT	Cuiaba	30,65	175360	78,60	MN428508
CB17	Serum	2018-03-11	2018-03-13	27	M	MT	Cuiaba	22,16	99926	83,90	MN428509
CB18	Serum	2018-04-24	2018-04-25	43	M	MT	Cuiaba	27,38	554552	79,90	MN428510
CB16	Serum	2018-04-12	2018-04-18	31	F	MT	Cuiaba	19,87	577642	85,10	MN428511
CB22	Serum	2018-03-10	2018-03-13	26	M	MT	Cuiaba	28,33	147381	88,50	MN428512
CB19	Serum	2018-04-05	2018-04-26	37	F	MT	Cuiaba	24,93	514308	82,70	MN428513
CB23	Serum	2018-03-13	2018-03-13	30	F	MT	Cuiaba	23,62	278973	53,00	MN428514
CB24	Serum	2018-03-18	2018-03-20	62	F	MT	Cuiaba	20,31	235010	93,50	MN428515
CB26	Serum	2018-03-21	2018-03-23	22	F	MT	Cuiaba	24,08	4167	86,50	MN428516
CB27	Serum	2018-03-15	2018-03-17	40	F	MT	Cuiaba	29,33	6855	84,10	MN428517
CB28	Serum	2018-03-07	2018-03-08	19	F	MT	Cuiaba	20,07	4391	89,40	MN428518
CB29	Serum	2018-03-15	2018-03-16	20	F	MT	Cuiaba	20,96	6384	88,20	MN428519
CB31	Serum	2018-03-08	2018-03-09	31	F	MT	Cuiaba	29,22	7537	85,20	MN428520
CB32	Serum	2018-03-16	2018-03-16	29	F	MT	Cuiaba	21,22	6010	87,10	MN428521
CB30	Serum	2018-03-07	2018-03-08	24	F	MT	Cuiaba	31,21	9104	83,50	MN428522
CB33	Serum	2018-03-08	2018-03-08	31	F	MT	Cuiaba	22,16	4753	89,40	MN428523
CB34	Serum	2018-03-05	2018-03-06	34	F	MT	Cuiaba	22,25	6554	86,50	MN428524
CB35	Serum	2018-03-05	2018-03-06	24	F	MT	Cuiaba	25,24	4454	89,40	MN428525
CB36	Serum	2018-03-06	2018-03-06	16	F	MT	Cuiaba	21,42	8795	86,40	MN428526
CB37	Serum	2018-03-05	2018-03-06	23	F	MT	Cuiaba	26,95	3957	89,40	MN428527

Ct = RT-qPCR quantification cycle threshold value.

### cDNA synthesis and multiplex tiling PCR

Samples were submitted to a cDNA synthesis protocol [11] using ProtoScript II First Strand cDNA Synthesis Kit. Then, a multiplex tiling PCR was conducted using Q5 High Fidelity Hot-Start DNA Polymerase (New England Biolabs) and a CHIKV sequencing primers scheme (divided into two separated pools) designed using Primal Scheme (http://primal.zibraproject.org) [12]. The thermocycling conditions involved 40 cycles, and reaction conditions was previously reported in [12].

### Library preparation and nanopore sequencing

Amplicons were purified using 1x AMPure XP Beads and cleaned-up PCR products concentrations were measured using Qubit dsDNA HS Assay Kit on a Qubit 3.0 fluorimeter (ThermoFisher). DNA library preparation was carried out using the Ligation Sequencing Kit and the Native Barcoding Kit (NBD104, Oxford Nanopore Technologies, Oxford, UK) [12]. Purified PCR products of each sample were quantified and DNA concentration were normalised before barcoding reactions. One barcode was used per sample in order to maximize the number of samples per flow cell. Sequencing library was loaded onto a R9.4 flow cell, and data was collected for up to 48 hours, but generally less.

### Generation of consensus sequences

Raw files were basecalled using Guppy and barcode demultiplexing was performed using qcat. Consensus sequences were generated by *de novo* assembling using Genome Detective (https://www.genomedetective.com/) [13].

### Phylogenetic and Bayesian analysis

The 24 new sequences reported in this study were initially submitted to a genotyping analysis using the phylogenetic arbovirus subtyping tool, available at http://genomedetective.com/app/typingtool/chikungunya [14]. After excluding low-quality genomes, > 10% of ambiguous positions, the newly genomic data generated in this study were aligned with 89 CHIKV-ECSA genome sequences from Brazil plus all available CHIKV-ECSA genome sequences from Americas (currently available only from Paraguay n = 5 and Haiti n = 2). Full details of the sequences used are provided in (**S1 Table**). Sequences were aligned using MAFFT [15] and edited using AliView [16]. The dataset was assessed for presence of phylogenetic signal by applying the likelihood mapping analysis implemented in the IQ-TREE 1.6.8 software [17]. A Maximum likelihood phylogeny was reconstructed using IQ-TREE 1.6.8 software under the HKY+G4 substitution model [17]. In order to investigate the temporal signal in our CHIKV-ECSA dataset we regressed root-to-tip genetic distances from this ML tree against sample collection dates using TempEst v 1.5.1 [18]. The ML phylogeny was used as a starting tree for Bayesian time-scaled phylogenetic analysis using BEAST 1.10.4 [19]. We employed a stringent model selection analysis using both path-sampling and steppingstone models to estimate the most appropriate model combination for Bayesian phylogenetic analysis [20]. The best fitting model was the HKY+G4 substitution model with a Bayesian skyline coalescent model [21, 22]. A discrete phylogeographical model *[23]* was also used to reconstruct the spatial diffusion of the virus across the compiled dataset sampling locations. Phylogeographic analyses were performed by applying an asymmetric model of location transitioning, coupled with the Bayesian stochastic search variable selection (BSSVS) procedure. Monte Carlo Markov chains (MCMC) were run for sufficiently long to ensure stationarity and an adequate effective sample size (ESS) of >200.

### Epidemiological data assembly

Data of weekly notified CHIKV cases in Brazil, availables at the Sistema de Informação de agravos de notificação (SINAN) (https://portalsinan.saude.gov.br/dados-epidemiologicos-sinan), were supplied by Brazilian Ministry of Health and were plotted using the R software version 3.5.1.

## Results

**Fig 1:**
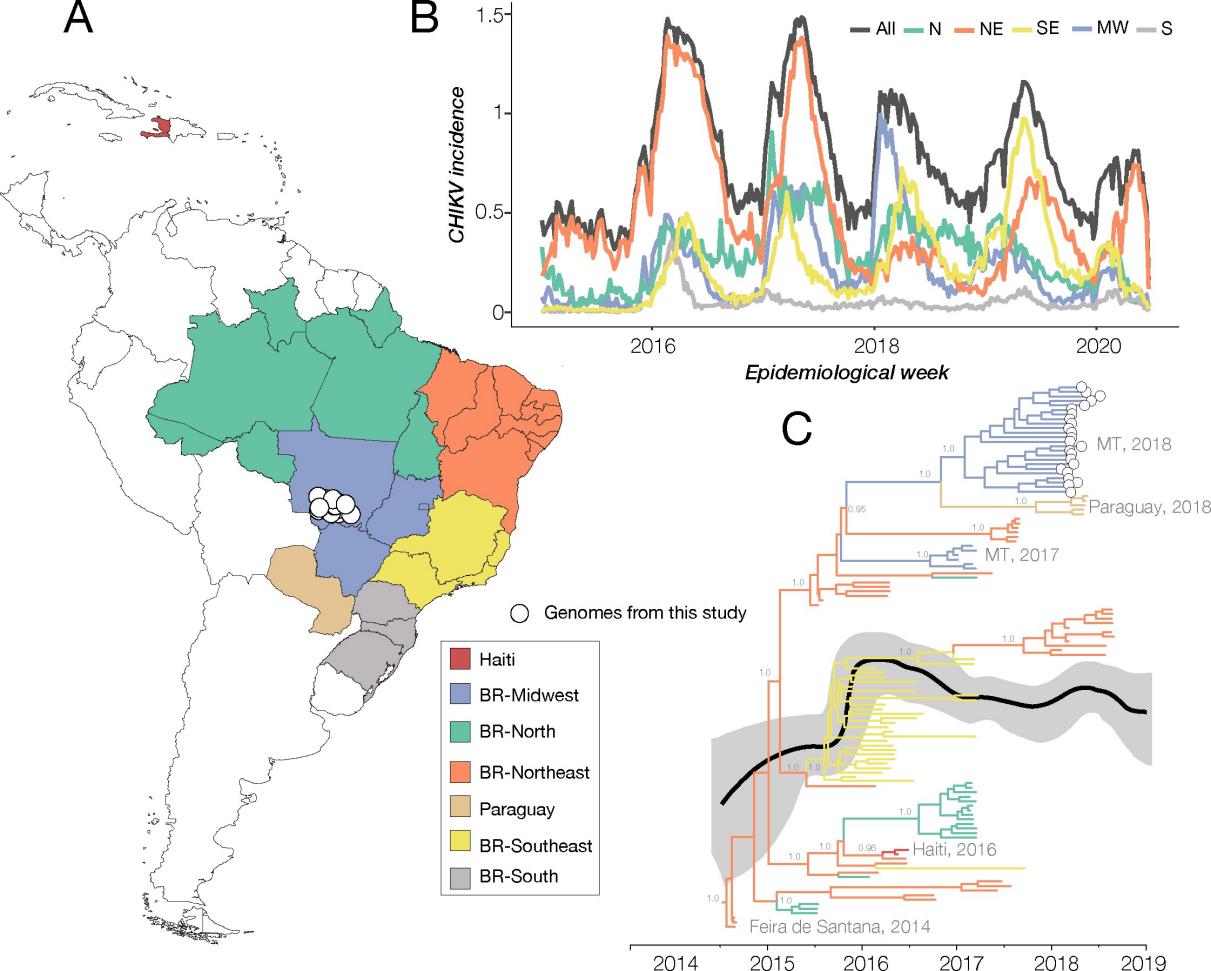
CHIKV transmission dynamics in Brazil (2014–2019). (A) Map of Brazil and Americas showing sampling location of the CHIKV genomes from this study (white circles). (B) Weekly notified chikungunya cases normalized per 100K individuals per Brazilian region in 2015–2020 (until EW06). Incidence (cases per 100K population) is presented in log10 for visual purposes. Epidemic curves are coloured according to geographical macro region: SE = Southeast, NE = Northeast, MW = Midwest, N = North, S = South. (C) Time-scaled phylogeographic tree of 120 complete and near-complete CHIKV genome sequences from the ECSA genotype sampled in Brazil and Americas. Colours represent different sampling locations according to the legend on the left of the tree. Tip circles (white) represent the genome sequences generated in this study. Skyline plot (black and gray lines) is superimposed. Relative genetic diversity is represented here as a surrogate for the product of effective population size and generation time. The solid black line represents the mean relative genetic diversity and the light gray area around the line represents the 95% HPD interval of the estimates.

We obtained CHIKV RT-qPCR positive clinical samples as part of the genomic surveillance project called ZIBRA 2 (https://www.zibra2project.org/), which aimed to perform, from a lab on wheels, genome sequencing of arboviruses circulating in Midwest Brazil, in the Mato Grosso state. Supported by the Brazilian Ministry of Health and PAHO/WHO, ZIBRA 2 has carried out genomic surveillance of arboviruses in the northern, northeastern and southeastern regions of Brazil [5, 6, 24]. We used the portable MinION sequencer and an amplicon approach [12] to generate 24 partial and near complete CHIKV genomes from serum samples provided by the Central Public Health Laboratory and collected during the 2018 outbreak in the midwestern state of Mato Grosso (MT). These samples, most of which were collected in March 2018, had an average Ct value of 24.48 (ranging from 18.59 to 31.21) and were from patients with average 29 years of age, and in their majority were female (87.5%) living in the city of Cuiaba, MT (**Table 1 and Fig 1A**).

The 24 new CHIKV genome sequences from MT had a mean genome coverage of 81.79% (coverage range 43.7%-93.6%). This genome coverage obtained is considered sufficient to perform phylogenetic inferences, according to a study that showed the occurrence of a decrease in phylogenetic accuracy when genome coverage is reduced from 40% to 20% [25].

Genomic data obtained in this study belonged to the ECSA lineage, as confirmed by the chikungunya virus typing tool (https://www.genomedetective.com/) [14], and clustered with other Brazilian sequences from previous outbreaks reported in other geographic regions (**Fig 1C**). These new genome sequences were submitted to GenBank under the accession numbers MN428504-MN428527.

**Fig 1B** shows the CHIKV weekly cases normalized per 100K individuals notified between 2015 and 2020 (until epidemiological week–EW 06) in five Brazilian regions: Southeast (SE), Northeast (NE), Midwest (MW), North (N), and South (S). Five CHIKV epidemic waves were found (2016–2020), characterized by a reduction in total cases per year from 2018 to 2020. Although without further information it is difficult to assert the drivers of this reduction in incidence, it is likely that significant herd-immunity has accumulated since CHIKV’s introduction, as suggested in other studies [26, 27]. Weekly reported incidence reveals that chikungunya was mostly reported in the Northeast region between 2015 and 2018, the South region had the lowest incidence in the entire time period, and that contrary to all other regions, the Southeast has presented an increasing incidence trend over the years (**Fig 1B**).

To explore in more detail the evolutionary relationship of these new MT sequences in the Brazilian context and to infer the ancestral location of CHIKV strains circulating in MT, we used a Bayesian discrete phylogeographic approach, employing the uncorrelated relaxed molecular clock and a Bayesian Skyline coalescent model (and a linear regression of root-to-tip genetic distance against sampling date which revealed sufficient temporal signal, r2 = 0.70; **S1 Fig**) in a dataset comprising the new MT sequences described in this study plus 96 published CHIKV genomes (**Fig 1C**). The Bayesian demographic reconstructions (**Fig 1C**, superimposed to the tree) provided evidence of seasonal oscillations, although with wide credible intervals, highlighting a slight but gradual decline of the median Effective population size (Ne) estimated over 2018–2020 which appears to be in agreement with decreasing incidence in time (**Fig 1B**).

Our Maximum clade credibility (MCC) tree showed that the new 2018 MT isolates formed a single well-supported clade, with posterior support of 1.0 (**Fig 1C**). Interestingly, these new isolates did not group with the other six previously published sequences sampled in 2017 also from Cuiabá-MT [28]. As these 2017 MT sequences also formed a well-supported clade (posterior support = 1.0), our MCC tree topology suggests that at least two independent introductions of the ECSA lineage occurred in MT. A previous study reported the circulation of CHIKV ECSA lineage in MT and indicated, by RT-qPCR, that the earliest human case of the disease recorded in that state dates to July 2015 [8]. From our time-measured tree, we estimated the time of the most recent common ancestor (TMRCA) of the two independent introduction events in MT to be between late September 2016 (May 2016 to December 2016, 95% HPD) for the first introduction event, and early February 2017 (September 2016 to June 2017, 95% HPD) for the second event. This interval does not include the earliest CHIKV positive case previously reported in MT and this divergence might display an absence of sufficient data because of non-sampling of earliest isolates [8].

The phylogeographic analysis showed that CHIKV was most likely introduced in MT from a dispersion event originating in the northeastern region of Brazil (location probability 0.98). Clades comprising other isolates from 2017–2018 and sampled in the northeastern region illustrate the persistence and re-emergence of the ECSA lineage in the northeastern region of Brazil since its introduction in 2014. In addition, our tree shows the 2018 MT outbreak clade is closely related to the clade containing isolates from Paraguay sampled in 2018, when 1,237 CHIKV cases were reported in that country [1]. We estimate that the TMRCA of the isolates from Paraguay dates back to December 2017 (June 2017 to May 2018, 95% HPD) and probably originated in the midwestern region of Brazil (location probability 0.90). This would be the second event of cross border transmission from Brazil, as our tree also shows isolates from Haiti sampled in 2016 clustered with an isolate from Northeast Brazil also sampled in 2016 (posterior support = 0.97, location probability 0.97). These results indicate a possible role of Brazil as a source for cross-border dispersion of the CHIKV ECSA lineage to other countries in the Americas since its introduction into the country.

## Discussion

More than 930,000 cases have been notified since CHIKV was first detected in Brazil in 2014 (4). Despite this large burden of disease, much is unknown about the origins of the virus responsible for the Brazilian outbreaks. To get more insight regarding CHIKV dispersion through different Brazilian regions and South American countries we generated 24 partial and near complete genome sequences from the 2018-CHIKV-ECSA epidemic registered in the state of Mato Grosso (MT), Midwest of Brazil, using a combined strategy of a mobile sequencing mission through this region, genomic, and epidemiological analysis.

Epidemiological data revealed yearly patterns of CHIKV transmission with a reduction in the number of reported cases over 2018–2020, likely a consequence of an expected, gradual accumulation of herd-immunity over the 7 years since its introduction in 2014. Phylogeographic reconstructions suggest that at least two independent introductions of the ECSA lineage occurred in MT from a dispersion event originating in the northeastern region of Brazil and estimated the time of the most recent common ancestor (TMRCA) to be between late September 2016 for the first introduction event, and early February 2017 for the second event. Furthermore, our analysis suggests that the midwestern Brazilian region appears to have acted as a source of virus transmission towards Paraguay, a bordering South American country to the Brazilian Midwest.

In summary, our data reveals a complex pattern of CHIKV transmission between epidemic seasons and sampled locations and suggests that Brazil has played a role as source for international dispersion (enhanced by cross-border transmission to other Americas countries such as Paraguay and Haiti) of the CHIKV-ECSA genotype to other American countries. Those results highlight the utility of combining genomic, epidemiological and evolutionary methods to understand ongoing mosquito-borne epidemics. Our analyses further indicate that additional data is required to better identify routes of CHIKV-ECSA genotype transmission into Brazil, and to understand its transmission dynamics through other American countries.

## Supporting information

S1 Fig Analysis of temporal structure in CHIKV-ECSA genotype. Root-to-tip genetic divergence against time of sampling. Colours represent different sampling locations.
(PDF)

S1 Table Full details of the 96 complete and near-complete CHIKV genome sequences from the ECSA genotype samples in Brazil and Americas used in this study. (DOCX)
