# Supplementary figures and images for: Short report: Introduction of chikungunya virus ECSA genotype into the Brazilian Midwest and its dispersion through the Americas
Source: PLoS Negl Trop Dis. 2021 Apr 16;15(4):e0009290. doi: 10.1371/journal.pntd.0009290 (PMC8051810; doi:10.1371/journal.pntd.0009290)

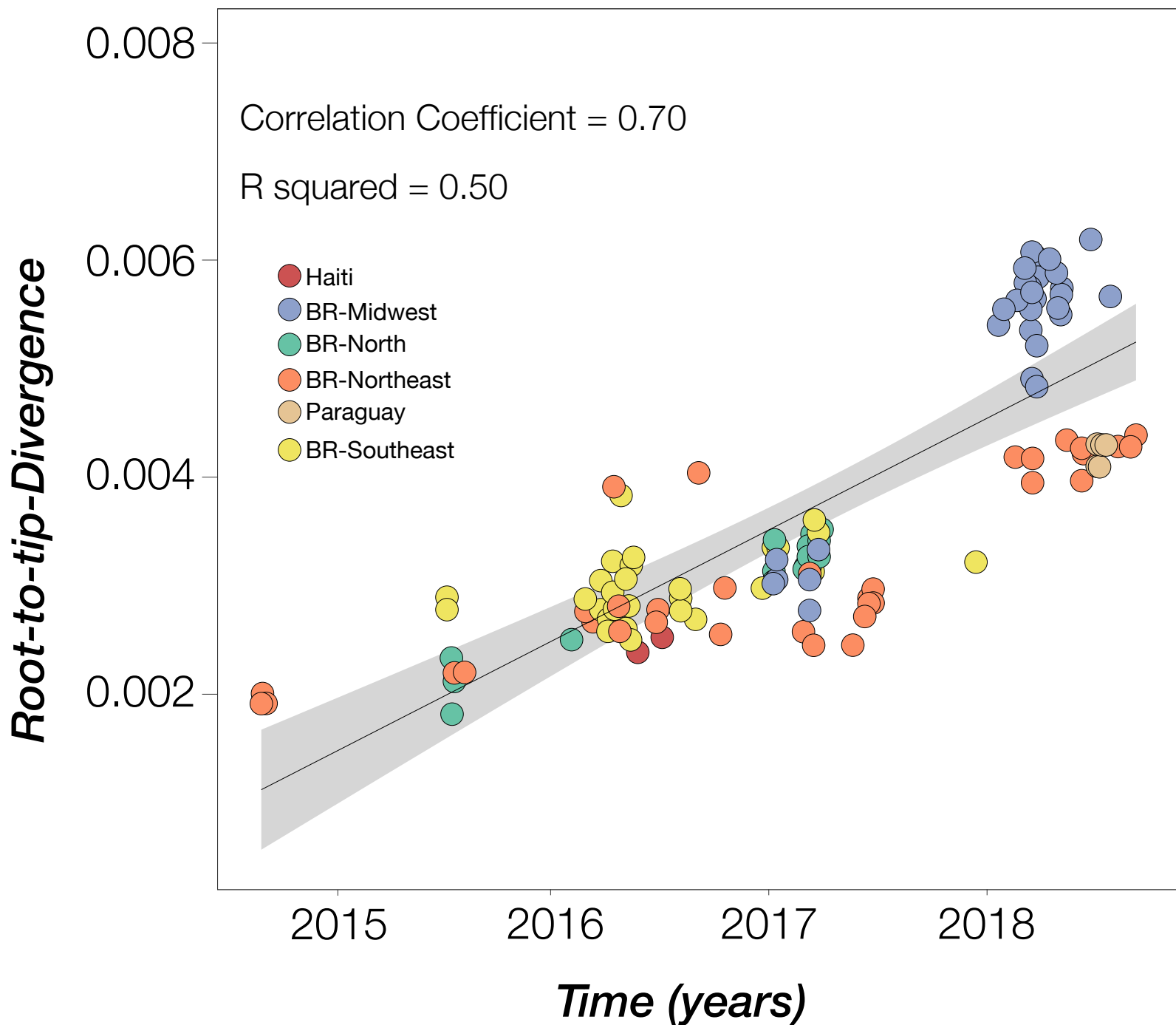

Supplement: S1 Fig — Root-to-tip genetic divergence against time of sampling. Colours represent different sampling locations. (PDF) [file pntd.0009290.s001.pdf]
