# Supplementary material for: Short report: Introduction of chikungunya virus ECSA genotype into the Brazilian Midwest and its dispersion through the Americas
Source: PLoS Negl Trop Dis. 2021 Apr 16;15(4):e0009290. doi: 10.1371/journal.pntd.0009290 (PMC8051810; doi:10.1371/journal.pntd.0009290)
Supplement: S1 Table — (DOCX) [file pntd.0009290.s002.docx]

**Supplementary Table 1**. Full details of the 96 complete and near-complete CHIKV genome sequences from the ECSA genotype samples in Brazil and Americas used in this study.

| **Acession Number** | **State** | **Country** | **Collection date** |
| --- | --- | --- | --- |
| MK121894 | Amazonas | Brazil | 27/01/2016 |
| MG649972 | Rio de Janeiro | Brazil | 05/05/2016 |
| MT038403 | Bella Vista | Paraguay | 10/07/2018 |
| MT038402 | Bella Vista | Paraguay | 03/07/2018 |
| MT038400 | Bella Vista | Paraguay | 29/06/2018 |
| MT038401 | Pedro Juan Caballero | Paraguay | 25/06/2018 |
| MT038399 | Pedro Juan Caballero | Paraguay | 25/06/2018 |
| MK752956 | Bahia | Brazil | 25/06/2018 |
| MK752952 | Bahia | Brazil | 30/08/2017 |
| MK752950 | Bahia | Brazil | 30/08/2017 |
| MK752958 | Bahia | Brazil | 23/08/2017 |
| MK752957 | Bahia | Brazil | 23/08/2017 |
| MK752953 | Bahia | Brazil | 16/08/2017 |
| MH823667 | Mato Grosso | Brazil | 01/03/2017 |
| MH823665 | Mato Grosso | Brazil | 16/03/2017 |
| MH823663 | Mato Grosso | Brazil | 05/01/2017 |
| MH823668 | Mato Grosso | Brazil | 05/01/2017 |
| MH823666 | Mato Grosso | Brazil | 01/03/2017 |
| MH823664 | Mato Grosso | Brazil | 16/03/2017 |
| MK518395 | Maranhao | Brazil | 16/05/2017 |
| MK121895 | Amazonas | Brazil | 18/03/2017 |
| KY704942 | Alagoas | Brazil | 14/04/2016 |
| KY704947 | Alagoas | Brazil | 15/04/2016 |
| KY704939 | Alagoas | Brazil | 17/04/2016 |
| KY704952 | Alagoas | Brazil | 07/04/2016 |
| KU940226 | Bahia | Brazil | 01/08/2015 |
| KU940225 | Bahia | Brazil | 15/07/2015 |
| MK156055 | Bahia | Brazil | 21/08/2018 |
| MK156054 | Bahia | Brazil | 24/08/2018 |
| MK156056 | Bahia | Brazil | 21/08/2018 |
| MK156053 | Bahia | Brazil | 24/08/2018 |
| MK156064 | Bahia | Brazil | 09/03/2018 |
| MK156062 | Bahia | Brazil | 20/08/2018 |
| MK156061 | Bahia | Brazil | 29/08/2018 |
| MK156063 | Bahia | Brazil | 09/03/2018 |
| MK156058 | Bahia | Brazil | 30/07/2018 |
| MK156060 | Bahia | Brazil | 08/02/2018 |
| MK156059 | Bahia | Brazil | 27/07/2018 |
| MK244641 | Rio de Janeiro | Brazil | 09/03/2017 |
| MK244640 | Rio de Janeiro | Brazil | 07/03/2017 |
| MK244635 | Rio de Janeiro | Brazil | 19/02/2016 |
| MG649983 | Rio de Janeiro | Brazil | 28/03/2016 |
| MG649970 | Rio de Janeiro | Brazil | 29/03/2016 |
| MG649977 | Rio de Janeiro | Brazil | 28/07/2016 |
| MG649984 | Rio de Janeiro | Brazil | 2015 |
| MG649975 | Rio de Janeiro | Brazil | 27/07/2016 |
| MG649982 | Rio de Janeiro | Brazil | 24/03/2017 |
| MG649978 | Rio de Janeiro | Brazil | 16/03/2017 |
| MG649976 | Rio de Janeiro | Brazil | 29/03/2016 |
| KY124328 | Rio de Janeiro | Brazil | 16/03/2016 |
| MG649971 | Rio de Janeiro | Brazil | 22/08/2016 |
| MG649981 | Rio de Janeiro | Brazil | 28/07/2016 |
| MG649974 | Rio de Janeiro | Brazil | 08/04/2016 |
| MG649980 | Rio de Janeiro | Brazil | 27/04/2016 |
| MG649979 | Rio de Janeiro | Brazil | 2017-03 |
| MK244632 | Rio de Janeiro | Brazil | 05/04/2016 |
| MK244634 | Rio de Janeiro | Brazil | 27/04/2016 |
| MK244637 | Rio de Janeiro | Brazil | 10/05/2016 |
| MK244636 | Rio de Janeiro | Brazil | 02/05/2016 |
| MK244633 | Rio de Janeiro | Brazil | 06/05/2016 |
| MK244638 | Rio de Janeiro | Brazil | 05/04/2016 |
| MK244639 | Rio de Janeiro | Brazil | 19/04/2016 |
| KY124329 | Rio de Janeiro | Brazil | 16/03/2016 |
| MG649985 | Rio de Janeiro | Brazil | 2015 |
| MG649973 | Rio de Janeiro | Brazil | 12/12/2016 |
| KY055011 | Sergipe | Brazil | 20/02/2016 |
| MK121907 | Roraima | Brazil | 27/02/2017 |
| MK121906 | Roraima | Brazil | 27/02/2017 |
| MK121899 | Roraima | Brazil | 17/03/2017 |
| MK121897 | Roraima | Brazil | 20/02/2017 |
| MK121903 | Roraima | Brazil | 15/03/2017 |
| MK121904 | Roraima | Brazil | 02/03/2017 |
| MK121898 | Roraima | Brazil | 22/02/2017 |
| MK121905 | Roraima | Brazil | 02/03/2017 |
| MK121908 | Roraima | Brazil | 05/03/2017 |
| MK121896 | Roraima | Brazil | 03/03/2017 |
| MK121900 | Roraima | Brazil | 17/03/2017 |
| MK121902 | Roraima | Brazil | 17/03/2017 |
| MK121901 | Roraima | Brazil | 17/03/2017 |
| MG000875 | Haiti | Haiti | 27/06/2016 |
| MG000876 | Haiti | Haiti | 17/05/2016 |
| KY704955 | Joao Pessoa | Brazil | 17/06/2016 |
| KY704954 | Joao Pessoa | Brazil | 20/06/2016 |
| KY704954 | Rio de Janeiro | Brazil | 18/02/2018 |
| KX228391 | Pernambuco | Brazil | 03/03/2016 |
| MK752954 | Bahia | Brazil | 22/06/2017 |
| MK752951 | Bahia | Brazil | 27/07/2017 |
| MK752955 | Bahia | Brazil | 05/06/2017 |
| MK159124 | Bahia | Brazil | 03/10/2016 |
| MK159127 | Bahia | Brazil | 10/10/2016 |
| MK121893 | Amazonas | Brazil | 15/07/2015 |
| MK121892 | Amazonas | Brazil | 11/07/2015 |
| MK121891 | Amazonas | Brazil | 10/07/2015 |
| KP164568 | Bahia | Brazil | 26/08/2014 |
| KP164570 | Bahia | Brazil | 03/09/2014 |
| KP164569 | Bahia | Brazil | 28/08/2014 |
